# Promoting Researchers and Policy-Makers Collaboration in Evidence-Informed Policy-Making in Nigeria: Outcome of a Two-Way Secondment Model between University and Health Ministry

**DOI:** 10.15171/ijhpm.2017.123

**Published:** 2017-10-22

**Authors:** Chigozie Jesse Uneke, Abel Ebeh Ezeoha, Henry Chukwuemeka Uro-Chukwu, Chinonyelum Thecla Ezeonu, Jonathan Igboji

**Affiliations:** ^1^African Institute for Health Policy & Health Systems, Ebonyi State University, Abakaliki, Nigeria.; ^2^Department of Medical Microbiology/Parasitology, Faculty of Medicine, Ebonyi State University, Abakaliki, Nigeria.; ^3^Department of Accountancy & Banking/Finance, Faculty of Management Sciences, Ebonyi State University, Abakaliki, Nigeria.; ^4^National Obstetrics Fistula Centre, Abakaliki, Nigeria.; ^5^Department of Paediatrics, Faculty of Medicine, Ebonyi State University, Abakaliki, Nigeria.; ^6^Ebonyi State Ministry of Health, Abakaliki, Nigeria.

**Keywords:** Researchers, Policy-makers, Collaboration, Evidence-Informed, Secondment, Nigeria

## Abstract

**Background:** There is need to strengthen institutions and mechanisms that can more systematically promote interactions between researchers, policy-makers and other stakeholders who can influence the uptake of research findings. In this article, we report the outcome of a two-way secondment model between Ebonyi State University (EBSU) and Ebonyi State Ministry of Health (ESMoH) in Nigeria as an innovative collaborative strategy to promote capacity enhancement for evidence-to-policy-to-action.

**Methods:** This study was an exploratory design with a quantitative cross-sectional survey technique. A secondment memorandum of understanding (MOU) was signed between heads of EBSU and ESMoH. The secondment program lasted six months with ten researchers and ten policy-makers spending up to two days per week in each other’s organization. The secondee researchers got engaged in policy-making and implementation activities in ESMoH, while the policy-maker secondees got involved in research activities in EBSU. Secondees evidence-to-policy capacity enhancement meetings were held and questionnaires designed in 5-point Likert scale were used to assess their impact.

**Results:** The secondee policy-makers and researchers admitted having considerable knowledge of secondment with mean ratings (MNRs) of 3.40 and 3.74 respectively on the 5 points scale. Secondment appeared to be more common in the policy-makers’ organization (MNRs: 2.80-3.07) than in the researchers’ institution (MNRs: 2.58-2.84). The secondee policy-makers participated in some academic and research activities including serving in research ethics committee in EBSU and provided policy-making perspective to the activities. The secondee researchers supported the policymaking process in ESMoH through policy advisory roles, and provided capacity enhancement for staff of the ministry on the use of research evidence in policy-making. There was a noteworthy increase on knowledge of policy analysis and contextualization among the secondees ranging from 20.7% to 50.4% and 31.3% to 42.8% respectively following a training session. A Society for Health Policy Research and Knowledge Translation was established by mutual agreement of secondees as a platform to permanently institutionalize the collaboration.

**Conclusion:** The outcome of this study clearly suggests that secondment has great potential in promoting evidence informed policy-making and merits further consideration.

## Background

Throughout the world there is a general consensus that a huge gap exists between policy-makers and researchers. This undeniable gap is known to be responsible for the problem of translating research evidence into policy.^1^ To address this challenge, there is need to strengthen institutions and mechanisms that can more systematically promote interactions between researchers, policy-makers and other stakeholders who can influence the uptake of research findings.^2^ Regardless of the subject under study, it is crucial for both researchers and policy-makers to recognize the value of coming together in what is in fact a symbiotic relationship – a relationship in which policy-makers and implementers generate feedback from the front lines, while researchers provide expertise in research methods needed for trustworthy studies.^3^ There is sufficient evidence showing that it is only by coming together in this way, that policy-makers and researchers can ensure that the knowledge generated is valid, and aligned with the health needs of society.^3-8^

The need for collaboration between policy-makers and researchers in the promotion of the evidence to policy link, then, is not in question. The difficulty lies in determining and establishing mechanisms of collaboration between these two key actors that will produce effective health policies and the most impactful health outcomes. According to Costello and Zumla,^9^ old models of research collaboration where data gathering by local researchers, and interpretation and publication by national or international researchers should be abandoned. The authors argued that new models are needed which can place building mutual trust and shared decision-making with clear national data ownership and development of research capacity across all stakeholders at the forefront.^9^ Based on the arguments of Costello and Zumla, we proposed a secondment mechanism between the university and the health ministry as a new model of partnership to promote capacity enhancement in evidence informed health policy-making and health policy implementation in Nigeria.

Secondment is defined as where an employee temporarily transfers to another job for a defined period of time for a specific purpose, to the mutual benefit of all parties.^10^ Secondments have been shown to offer the opportunity to enhance personal development and working practices for front-line staff through valuable first-hand encounters whereby the secondee will experience new concepts, values and cultures which can test their ability to succeed in a different environment.^11^ Secondments therefore can provide a positive way of motivating people and increasing work satisfaction, whilst enhancing best practice, collaborative working partnerships, knowledge and skills.^11^ The practice of secondment is generally known as a strategy for skills development for mutual organizational and individual benefit, but there is, however, a gap in the literature regarding practical implementation considerations and critical success factors related to the use of secondment as a global health policy development and implementation strategy.^12^

The literature is very scarce especially from low- and middle-income countries (LMICs) on the use of secondments between an organization that is in position to produce research evidence (university) and the one that is in position to make use of evidence (such as the health ministry) to enhance evidence-informed policy-making and policy implementation. In this article, we report the outcome of a two-way secondment model between Ebonyi State University (EBSU) and Ebonyi State Ministry of Health (ESMoH) in Nigeria as an innovative collaborative strategy to promote capacity enhancement for evidence-to-policy-to-action.

## Methods

### Study Design and the Rationale

This study was designed as an exploratory investigation with a quantitative cross-sectional survey technique. We employed this design based on the experience and lessons we learnt from our previous exploratory study on individual and organizational capacity for evidence use in policy making in Nigeria.^13^ Our focus was to gain understanding and insights that would facilitate the development of a more robust future definitive scientific investigation. Some previous reports have supported the use of exploratory research as a strategy that can engender improved understanding of a concept or to help crystallise the definition of a problem and to identify important variables to be studied.^14-16^

Our approach is supported by Asadoorian and colleagues,^15^ who argued that exploratory research was important in contributing to understanding the cultural practice milieu in relation to individual characteristics in implementing evidence into practice with the overall aim of improving healthcare delivery and outcomes. The quantitative cross-sectional component of our design has been described by previous workers as one of the ideal techniques for a preliminary investigation designed to provide baseline information that will aid the development of a more complex study strategy.^17-19^ To enable us to gain a deeper insight on the impact of the secondment programme, we added a qualitative methodology which provided opportunity to conduct a brief interview with selected researcher and policy-maker secondees. The interview was designed to determine the post-secondment perceptions of the secondees regarding impact of the programme.

Our choice of the two-way secondment strategy as a collaborative mechanism to enhance policy-makers’ and researchers’ capacity for implementation research is based on available report by the British Academy for Humanities & Social Sciences,^20^ which indicated that two-way secondments are an important means of facilitating dialogue and exchange between researchers and policy-makers. The report noted that secondments can help researchers and policy-makers to develop understandings and connections that facilitate both knowledge transfer and policy impact.^20^ The secondment programme was categorized into three phases (pre-secondment, secondment, and post-secondment).

### Pre-secondment Phase

#### (i) Development and Signing of Secondment Agreement (Memorandum of Understanding) Between the University and Health Ministry

We developed a secondment agreement/memorandum of understanding (MOU) document following extensive consultation with relevant senior officials of EBSU and ESMoH. The MOU set out the legal liabilities of each party and also the practical arrangements for the secondment (Table 1). The document was signed by the Vice-Chancellor of EBSU and the Health Commissioner of ESMoH. The pre-secondment phase took four months to finalize (December 2015 to February 2016) and involved diplomatic interactions between the university and the Ministry of Health (MoH) before an agreement was reached. The secondment activities commenced following the endorsement of the MOU and lasted for 6 months (March 2016 to August 2016). The facilitators of the project were among the secondees. In line with the secondment agreement between the two organizations, all secondees continued to be on the payroll of their parent organizations (Table 1 item 4). The host organizations therefore did not provide any monetary payment to the secondees but granted them free access to facilities such as library, work/meeting space, internet access, organization subscribed platforms and databases and refreshments during meetings/workshops. The facilitators were paid stipends according to their level of effort at the various stages of the project which was provided by the project funder.

**Table 1:** Components of the Secondment Memorandum of Understanding Between EBSU and ESMoH

**S/No.**	**Secondment Parameter**	**Description of Secondment Agreement Terms**
1	The nature of the secondment	Part-time model
2	The amount of time the secondee will commit to the secondment	Up to two working days in per week
3	The duration of the secondment	Six months
4	The payment of the secondee	The home organizations will continue the payment of their staff on secondment
5	Who will manage the secondee during the secondment	To be handled by university Director of research & the Health Ministry Personnel Manager
6	How disciplinary or grievance issues will be dealt with	To be handled by university Director of research & the Health Ministry Personnel Manager
7	Mutual indemnities in respect of any acts/omissions of the employer or the host organization that lead to claims by the secondee	To be handled by university Director of research & the Health Ministry Personnel Manager
8	How each side may terminate the agreement including in what circumstances	To be handled by university Director of research & the Health Ministry Personnel Manager
9	Signatories of the secondment MOU	The Vice-Chancellor of the university and Commissioner of the health ministry

Abbrevaitions: MOU‏, memorandum‏ of‏ understanding‏; EBSU, Ebonyi State University; ESMoH, Ebonyi State Ministry of Health‏.

The facilitators of the secondment consisted of four senior researchers (two professors and two senior lecturers) from the university and two senior policy-makers (directors) from the MoH. The facilitators are involved together in an existing collaboration known as the Health Policy & Systems Research Project and were members of a health policy advisory committee commissioned by ESMoH in the state. They have worked together in a number of previous successful health policy projects involving the engagement of both policy-makers and researchers. The secondment programme was an initiative resulting from recommendations of previous projects undertaken by the facilitators.

#### (ii) Selection of Secondment Programme Participants

We consulted with the Commissioner for health of the ESMoH regarding the selection of the policy-maker secondees from the health ministry. The criteria for the selection of secondees included were as follows: secondee must be, (*i*) a senior staff of director cadre in the ESMoH, (*ii*) directly involved with policy-making/implementation process, (*iii*) willing to participate fully in the secondment programme. The participation of 10 policy-makers who fulfilled these criteria was approved by the Commissioner for health. A total of 10 Senior researchers from EBSU constituted the Secondee researchers. These included three members of the project team and seven others from the university. All secondee researchers were leaders of registered research teams, occupying senior academic positions in the university and indicated their willingness to participate in the program. Specific efforts were made to match the seniority of the secondees with their hosts.

#### (iii) Pre-secondment Sensitization Meeting

We organized a one-day pre-secondment programme meeting for all secondees. The meeting was interactive and used to sensitize the secondees regarding the MOU and other vital information regarding the secondment. A questionnaire designed in 5-point Likert scale (1-strongly disagree to 5-strongly agree) was also administered to assess the knowledge and perception of the participants regarding secondment in their organizations. This questionnaire used was initially pretested and standardized.

### Secondment Phase

#### (i) Researcher Secondees Activities at the Ministry of Health

The researcher secondees from the University provided technical support to the various units and departments of the health ministry particularly the State Malaria Elimination Programme and reproductive health services, and primary health care. Their terms of reference were as follows: (*i*) build trust and understand policy-maker’s evidence needs; (*ii*) play expert advisory role and provide scientific evidence to guide on policy issues; (*iii*) provide capacity enhancement for policy-makers.

### (ii) Policy-Maker Secondees Activities at the University

The policy-maker secondees from the MoH were integrated into some research and training activities in the university so they can bring in their policy-making perspectives in the academic activities in the university. They were involved in the following: (*i*) working with research groups; (*ii*) participation as course facilitators; (*iii*) serve as members of University Research Ethics Committee (UREC).

### Post-secondment Phase

### (i) Policy Contextualization and Policy Dialogue Event

The post-secondment phase commenced immediately after the 6-month duration of the secondment phase. We organized a one-day policy review/contextualization programme with all the secondees and other senior policy-makers of the health ministry in attendance in September 2016. The meeting was held at the MoH and used to produce a policy brief on malaria control using insecticide treated nets in Ebonyi State. During the workshop, we conducted training of secondees on writing skills, summarizing and policy briefing for disease control and also for policy analysis and policy review process. A policy dialogue was undertaken on the “National guidelines for the implementation of continuous distribution systems for the delivery of long lasting insecticidal nets through routine and other channels in Nigeria” (https://www.k4health.org/sites/default/files/ng.ta_.1_national_guidelines_on_continous_distribution_0.pdf). Participants deliberated on the national policy documents to identify the recommendations from the policy that can be applicable to the local context of Ebonyi State. Context specific net distribution approaches were determined for the State in order of priority including the implementation facilitators, barriers and strategies. We used anonymous self-administered questionnaires for the evaluation of the programme. The same questionnaire was used at the beginning of the programme as at the end, in order to be able to assess changes in participants’ knowledge. The questionnaire we employed have been used in previous similar study.^13^

### (ii) Initiating the Establishment of a Civil Society Organization as Permanent Platform for Collaboration

As part of effort to sustain the partnership between the policy-makers and researchers, the secondees agreed to initiate the establishment of a civil society organization that will serve as a platform to permanently bridge the gap between researchers and policy-makers and promote evidence-informed policy making in Nigeria.

### (iii) Comments From Selected Secondees on Impact of the Secondment on Their Commitment to Evidence to Policy Process

Three secondee policy-makers and three secondee researchers were asked to comment on how the secondment experience has improved their understanding and commitment to evidence-informed policy-making in the Nigerian context. An interview guide was used with the question *“How has the secondment programme improved your understanding about evidence-informed policy-making and how has the capacity acquired helped you to promote evidence to policy link in your organization?”*

### Data Analysis

The data collected via the questionnaire was analyzed using the methods developed at McMaster University, Hamilton, ON, Canada by Johnson and Lavis.^22^ The analysis is based on mean rating (MNR), median rating (MDR) and range. For instance, the figures represent Likert rating scale of 1-5 points, where 1 point = grossly inadequate; 2 points = inadequate; 3 points = fairly adequate; and 5 points = very adequate. In terms of analysis, values ranging from 1.00-2.99 points are considered low, whereas values ranging from 3.00-5.00 points considered high.

## Results

### Key Success Factor of the Secondment

The major success factor of the secondment programme was the already existing cordial working relationship among the secondees prior to the commencement of the secondment. A number of the researchers and policy-makers who participated in this programme have had opportunity of collaborating in previous health policy projects in the state and have served together in health committees.

### Outcome of the Secondment Programme in Terms of Comparative Knowledge and Involvement of Policy-Makers and Researchers

Both policy-makers and researchers admitted having fairly adequate to adequate knowledge of what secondment is all about with MNRs of 3.40 and 3.74 respectively (Table 2). Although the extent of implementation of secondment programmes among participants’ organizations was low, secondment appeared to be more common in the policy-makers’ organization (MNRs ranged from 2.80-3.07) than in the researchers’ institution (MNRs ranged from 2.58-2.84). Both policy-makers and researchers strongly agreed that secondments offer the opportunity to enhance personal development and working practices, and should be implemented on a continuous basis. They agreed that a continued secondment programme between the university and health ministry would enhance the partnership and promote the use of evidence in policy-making and, at the same time, would enhance capability development, understanding of context, and finding applications of research and enable more effective problem solving. They also agreed that a continued secondment programme will promote strong and sustained collaboration and can help eliminate mutual mistrust between policy-makers and researchers. The MNRs on these parameters ranged from 4.67-4.87 for policy-makers and 4.37-4.79 for the researchers on the scale of 5 (Table 2).

**Table 2:** Outcome of Comparative Knowledge and Involvement of Policy-Makers and Researchers Regarding Secondment Programme Between the University and Health Ministry Conducted at Pre-secondment Sensitization Meeting in Ebonyi State Nigeria

**Parameter Assessed**	**Mean Rating** **Policy-Makers**	**Mean Rating** **Researchers**	**Mean** **Difference**	**Percentage** **Mean Difference**
**Questions on Secondment With Ministry of Health or Other Policy-Making Organizations**				
(i). Knowledge of what secondment is all about.	3.40	3.74	0.34	10.00
(ii). Extent of implementation and execution of staff secondment among various units/ departments in your institution.	3.07	2.84	0.23	8.10
(iii). Extent of implementation and execution of staff secondment between your institution and other government ministries and agencies.	2.80	2.58	0.22	8.53
(iv). Extent of implementation and execution of staff secondment between your institution and ministry of health/university.	2.87	2.68	0.19	7.09
(v). Extent of agreement that Secondments offer the opportunity to enhance personal development and working practices whereby the secondee will experience new concepts, values and cultures which can test their ability to succeed in a different environment.	4.67	4.37	0.30	6.86
(vi). Extent of agreement that Secondment between the university and health ministry is worthwhile and should be implemented on continuous basis.	4.73	4.63	0.10	2.16
(vii). Extent of agreement that Secondment between the university and health ministry will improve partnership and promote the use of evidence in policy-making.	4.80	4.79	0.02	0.42
(viii). Extent of agreement that a two-way secondment model will enhance capability development, understanding context, finding applications/problem solving in disease control by the participating researchers and the policy-makers.	4.73	4.63	0.10	2.16
(ix). Extent of agreement that a secondment programme between the University and Health Ministry will promote strong and sustained collaboration at organizational level between both organizations.	4.73	4.68	0.05	1.07
(x). Extent of agreement that a secondment programme between the University and Health Ministry can help eliminate mutual mistrust and enhance personal development and partnership between researchers and policy-makers.	4.87	4.68	0.19	4.06
(xi). Extent of agreement that a secondment programme between the University and Health Ministry can motivate secondee policy-makers to acquire access to capabilities and facilities in the University, which are useful in evidence-informed policy-making.	4.80	4.63	0.17	3.67

### Outcome of Researcher Secondees Activities at the Ministry of Health

*(i) Build trust and understand policy-maker’s evidence needs:*

The researchers constantly interacted with the policy-makers during meetings and programmes at the MoH and also at other fora and were able to build trust. This helped the researchers to understand the richer and deeper context for their research as it relates to tailoring the research findings to meet the needs of the policy-makers. This gave them insights into how policy-makers use information and how information can be most effectively presented to them.

*(ii) Play expert advisory role and provide scientific evidence to guide on policy issues:* The researchers also supported the policy-making process through policy advisory role, and strategic analyses of high profile challenging policy issues. One key example was the policy review meeting on use of insecticide treated nets for malaria control in Ebonyi State. The researchers helped to guide the ministry of help to identify evidence-informed strategies that enabled the development of a policy brief with recommendations that were most suitable for Ebonyi State.

*(iii) Provide capacity enhancement for policy-makers:* The researchers used their broad technical and professional knowledge to provide capacity enhancement for staff of the ministry on how to assess and use evidence in policy-making and how to design policy relevant studies. This was accomplished at organized meetings and workshops at the MoH.

### Outcome of Policy-Maker Secondees Activities at the University

*(i) Working with research groups:* The policy-makers were attached to three research groups in the University working on malaria, schistosomiasis, child health, and women health. Their involvement in research team meetings, training sessions and data collection activities helped to increase their understanding and appreciation of the research process. They also acquired new capabilities and research skills especially those of long-term and strategic significance.

*(ii) Participation as course facilitators:* The policy-makers were engaged as facilitators in some of the course topics in the University three-month Certificate training programme on health policy/systems. This enabled them have opportunities to impart knowledge on their actual experience working in policy-making environment.

*(iii) Policy-makers as members of UREC:* Three of the policy-makers were appointed as members of the UREC. Their membership of the UREC enabled them to bring in the policy-maker’s perspectives into the assessment of research project for ethical clearance. Among the critical issues they raised at the UREC meetings was the need for research proposals submitted to the committee to be tailored towards policy so as to enhance uptake by policy-makers.

### Outcome of Policy Review/Contextualization Program

The outcome of the analysis of the pre-program and post-program questionnaire from the policy review/contextualization training session indicated a tremeondous improvement in the knowledge and understanding of the topics taught as shown by noteworthy increase in the mean rating percentages in each topic (Table 3). The range of percentage increase in the mean ratings for each topic is as follows: Knowledge of meaning of policy (9.92%-22.96%); Knowledge of policy review process (31.29%-42.76%); Knowledge of policy analysis (20.23%-46.62%). Regarding the overall assessment of the training workshop, 53.57% of participants scored the workshop 61%-80%, while 39.29% score the workshop 81%-100%.

**Table 3:** Outcome of the Pre-workshop and Post-workshop Questionnaire Analyais for Policy Review/Contextualization Training Workshop in ESMoH, Nigeria

**Parameters assessed**	**Pre-workshop Mean**	**Post-workshop Mean**	**Mean** **Increase**	**% Mean Increase**
**Knowledge of Meaning of Policy **				
1. What is your level of knowledge of the meaning and elements of policy?	3.83	4.21	0.38	9.92
2. How would you rate your understanding of policy cycle?	3.31	4.07	0.76	22.96
3. What is your level of understanding of the concept of policy process and policy assistance?	3.62	4.17	0.55	15.19
4. What is your level of understanding of role of interests, ideology and values?	3.48	3.79	0.31	8.91
**Knowledge of Policy Analysis**				
1. What is your level of understanding of the Framework for an institutional analysis of policy processes?	2.66	3.90	1.24	46.62
2. What is your level of understanding of Analytical framework for context, evidence and links?	3.07	3.89	0.82	26.71
3. How would you rate your understanding of critical policy issues and the focus/forms of policy analysis?	2.97	3.89	0.92	30.98
4. What is your level of understanding of the concept of Policy making and use of evidence?	3.41	4.10	0.69	20.23
5. What is your level of understanding of the concept of Policy analyses?	3.28	3.96	0.68	20.73
**Knowledge of Policy Review Process **				
1. How would you rate your understanding of Success Factor Country Multi-stakeholder Policy Review methods?	2.69	3.83	1.14	42.38
2. What is your level of knowledge of Organizing the multi-stakeholder review?	2.79	3.97	1.00	35.84
3. How would you rate your understanding of policy review with respect to geographical context for health and development?	2.93	3.86	0.93	31.74
4. How would you rate your understanding of policy review with respect to Key trends, timelines and challenges?	2.83	4.04	1.21	42.76
5. How would you rate your understanding of policy review with respect to Health sector initiatives and investments?	2.97	3.93	0.96	32.32
6. How would you rate your understanding of policy review with respect to Initiatives/ investments in sectors outside of health?	2.90	3.89	0.99	34.14
7. How would you rate your understanding of policy review with respect to Key actors and political economy?	2.93	4.00	1.07	36.52
8. How would you rate your understanding of policy review with respect to Governance and leadership?	3.10	4.07	0.97	31.29

Abbreviation: ESMoH, Ebonyi State Ministry of Health.

The outcome of the policy dialogue is summarized in Table 4. The dialogue focused on the use of insecticide treated nets in the control of malaria in the rural areas of Ebonyi State. Both policy-makers and researchers deliberated extensively and selected three implementation approaches as follows: (*i*) Community Directed Distribution, (*ii*) School Based Systems, (*iii*) Health Facility Based. The facilitators and barriers to these approaches as well as the ideal strategies for their implementation that is specific to Ebonyi State as identified by participants are outlined in Table 4. Among the strategies recommended by the participants that will improve the implementation of the insecticide treated nets include advocacy to opinion leaders and community involvement, as well as engaging peer educators and establishing effective monitoring and evaluation by the MoH.

**Table 4:** Outcome of Policy-Makers/Researchers Policy Dialogue at the Policy Review/Contextualization Program a the ESMoH, Nigeria

**Policy Approach in Order of Priority**	**Facilitators**	**Barriers**	**Strategies**
Community Directed Distribution	1. Higher chance of acceptance by end users 2. Wider reach 3. Accessibility 4. Less cultural barrier 5. Less cost 6. Task shifting	1. Political interference 2. Living pattern (settlement pattern) 3. Lack of commitment from the CDDs 4. Lack of supervision 5. Inadequate knowledge 6. Sustainability challenges due to lack of motivation 7. System may open up corruption and abuse	1. Advocacy to opinion leaders 2. Community dialogue 3. Community participation 4. Raise community structure 5. Train community personnel 6. Use local language
School Based Systems	1. Coverage 2. Acceptability 3. Spillover effect 4. Sustainable 5. Cost effective 6. “Catch them early”	1. Difficulty in passing the message 2. Rejection by parents 3. Ethical issues 4. Limited supply time	1. PTA involvement 2. Parent consent 3. School based management committees 4. Peer educators 5. Teachers involvement 6. Appropriate communication methods like role play
Health Facility Based	1. Reach vulnerable group 2. Cost effective 3. Encourage demand for health care 4. Availability of qualified and skilful workers 5. Devoid of political manipulation 6. Building the capacity of health workers	1. Diversion of health workers’ duty-task shifting 2. Low coverage 3. Low perception of quality of healthcare workers 3. Unavailability of the commodity at health facility	1. Strengthen operating procedure 2. Reduce waste of manpower 3. Enhanced monitoring and evaluation by the ministry of health

Abbreviations: CDDs, Community development districts; PTA, Parents-teachers association; ESMoH, Ebonyi State Ministry of Health.

### Establishment of Society for Health Policy Research and Knowledge Translation

The Society for Health Policy Research and Knowledge Translation was established by mutual agreement of secondees from both the university and MoH. The initiative for the establishment of the Society came from the experiences of the secondment. The secondees desired a platform to continue to collaborate since the secondment programme had a life span. The Society therefore provided the platform for continuous networking. The Society was formally approved and registered by the Nigerian Federal Government through the Corporate Affairs Commission Abuja in October 2016. A meeting for the launching of the Society was held in November 2016 with the inauguration of a five-member board of trustees comprising of three senior researcher secondees from the university and two senior policy-makers from the MoH. The mission of the society is to promote multidisciplinary and intersectoral approaches that are evidence-informed for addressing societal health and developmental challenges leading to effective and impactful health policies.

### Comments From Secondees on Impact of the Secondment on Their Commitment to Evidence to Policy Process

Commenting on the impact of the programme, one of the policy-makers interviewed stated thus:

“*My involvement has revealed the inevitable need for evidence-informed policy much more than ever before, since before now I looked at research findings as purely an academic exercise.*”

This statement by the policy-maker indicates a change of mindset towards research evidence and suggests the policy-maker now considers research as very critical to the policy-making process. Another policy-maker confirmed this noting that:

“*My involvement in the program has affected my worldview on issues of rendering health services to people based more on scientific evidence rather than colloquial evidence.*”

The third policy-maker interviewed noted that he had started promoting the use of evidence in the policy-making process in his work at the MoH. He stated thus:

“*I have positively used the wealth of knowledge and experience I gained from the programme to influence evidence-informed Child Survival Health Policy in the MoH. The programme is highly commendable.*”

The secondee researchers interviewed generally noted that the programme helped them to realize the need to begin active collaboration with the policy-makers to enhance research to policy link. The first researchers noted thus:

“*This program has helped me to appreciate the demerits and merits of existing policies in our health systems and the need for researchers like us to help close existing gaps created by ignorance and poor communication with policy-makers.*”

Another researcher collaborated this by stating that:

“*The program has gone a long way towards enhancing the collaboration and encouraging effective interaction between researchers and policy-makers. The unwarranted complex that used to hinder cooperation between the two groups has since disappeared; and the result is the very interesting testimonies flowing from the policy-makers themselves about how feasible and viable the policy-making process has become.*”

The third researcher clearly indicated her willingness to commit to effective and continuous partnership with the policy-makers in the interest of the health sector. She noted that:

“*This program is an outstanding evidence to policy innovation, I now see policy-makers as indispensable partners in progress in the formulation of effective health policy and will continue to work with them.*”

## Discussion

To the best of our knowledge, this study was the first systematic implementation of a secondment program between a university and MoH in West Africa. Our overriding goal was to build a robust permanent partnership through secondment, in which both researchers and policy-makers can view themselves as partners in progress rather than rivals. The outcome of our assessment of the participants’ perception regarding secondment in their organization showed that secondment was a more common event in the health ministry than in the university. This was not unexpected because in Nigeria, secondment is an integral part of administrative procedure in all government ministries.^23^ Secondment is among the three principal ways appointments are made into government ministries or the civil service in Nigeria, the other two ways are through recruitment and transfers.^23,24^ To the secondee policy-makers, the secondment programme was not new as they are already very conversant with such a process, but experiencing it in an academic environment was to them an entirely new dimension to secondment. A previous report from Nigeria indicated the existence of some form of secondment between the university and industrial sector,^25^ but similar studies between the university and health ministry in Nigeria are essentially lacking. In a recent report on the use of secondments as a tool to increase knowledge translation, the policy-maker secondee to a university, became more aware of academic resources and recognised how secondments could be used to strengthen knowledge translation.^26^

The secondment program of this study, provided benefits not only to the secondees but also enhanced partnership between the university and MoH. The secondee policy-makers had opportunity of working in a research environment while the secondee researchers had opportunity to get exposed to policy-making environment thereby improving their perception of the operations of both organizations. Findings from a previous report have been shown that secondment can offer the opportunity to enhance personal development and working practices for front-line staff through valuable first-hand encounters whereby the secondee will experience new concepts, values and cultures which can test their ability to succeed in a different environment.^11^

In this study, the secondee policy-makers had the opportunity of getting involved in some academic and research activities going on in the university such as participating in some research projects, handling some courses in the university as course lecturers and participating in UREC. Also the secondee researchers had opportunity in participating in policy review process and provided technical support from research perspective to the MoH. Previous reports indicated that this type of secondment activity can provide a positive way of motivating people and increasing work satisfaction, whilst enhancing best practice, collaborative working partnerships, knowledge and skills.^4,27^ Although the practice of secondment is generally known as a strategy for skills development for mutual organizational and individual benefit, there is, however, a gap in the literature regarding practical implementation considerations and critical success factors related to the use of secondment as a global health policy development and implementation strategy.^9,28^

We explored some sustainable practical implementation considerations especially with more emphasis on policy-makers’ engagement. This was part of effort to bridge information gap on the use of secondment to enhance evidence to policy link. In addition to the training we introduced for capacity enhancement on policy review, contextualization and dialogue, we also introduced mechanisms that will more permanently involve the policy-makers on research matters in the university. These mechanisms included: working with research groups, participation as course facilitators and serving as members of UREC. A number of previous studies have reported that this level of engagement gives policy-makers opportunity to develop knowledge of research processes, and enable researchers to gain a better understanding of what research is relevant to policy-makers and how to communicate their findings.^26,29,30^ We used this strategy to strongly promote the ‘exchange’ model of KT which emphasises human interaction, with researchers and knowledge users coproducing research and disseminating results while at the same time strengthening the ‘pull’ model which stresses policy-maker’ needs-driven research.^31,32^

One of the outstanding features of this secondment program was the exposure of the secondees to policy analysis, contextualization and dialogue. To some of them it was their first experience of having a capacity enhancement training on policy analysis and contextualization. There was considerable improvement on participants’ knowledge as demonstrated by the post-program analysis. The percentage increase on knowledge of policy analysis and contextualization ranged from 20.7% to 50.4% and 31.3% to 42.8% respectively. The importance of health policy analysis as an instrument that can facilitate evidence to policy process is not in question as this is widely acknowledged in earlier reports.^33-35^ We strongly believe that the improved understanding of policy analysis by the secondees especially the policy-makers will help them consider policy context (political, economic and socio-cultural) from evidence perspective to influence the health policy process.

Another important dimension to this study was the inclusion of a policy dialogue which we believed will strengthen the secondees capacity for evidence-informed policy-making as noted in our previous reports.^36-38^ The policy dialogue which centred on a national policy on malaria control afforded the secondees opportunity to exchange knowledge and experience in order to have the best possible implementation options in the context of Ebonyi State. The policy dialogues allowed research evidence to be considered together with the views, experiences and tacit knowledge of the secondees. Lavis and colleagues,^39^ noted that policy dialogues represent a new and evolving approach to supporting evidence-informed policy-making and they are one of many forms of political interaction that could usefully be more evidence-informed. Furthermore, Lomas and colleagues,^40^ had earlier described policy dialogue as a deliberative process that is an effective tool for generating evidence-based, context sensitive guidance, and they point to design features that are likely to be successful.

The establishment of the Society for Health Policy Research and Knowledge Translation was a key success factor of our secondment program. It serves as an example of a platform that will provide a ‘longer term’ bridge of the gap between policy-makers and researchers and promote evidence to policy process. The interesting thing about the society is that it was jointly initiated by both policy-makers and researchers involved in our secondment program. The establishment of the Society signifies the institutionalization of policy-makers’-researchers’ alliance as an intrinsic part of evidence-informed policy-making. There is no doubt that the society will serve as a mechanism that will continue to drive evidence-informed policy-making and knowledge translation in Nigeria as exemplified by our previous similar initiative (health policy advisory committee).^36,41^ The comments of the secondees clearly showed the secondment program had a significant impact on them and the willingness and commitment to the partnership were very evident.

## Conclusion

This study was not without some limitations. First, the secondment period was rather too short to assess any major impact the program had on evidence-informed policy-making process in the State. Second, we acknowledge the fact that our study design ie, exploratory investigation with a quantitative cross-sectional component was limited and a more complex study design is recommended for future study. A third limitation was the use of self-assessment technique to evaluate the training outcomes. Deans and Ademokun,^42^ highlighting the weakness of this technique noted that being able to critically recognize and understand one’s own gap in skills and knowledge is a difficult process which takes guided thought. A strength of the project was in the inclusion of observation and qualitative information to it alongside the numerical data, enabling cautious interpretation. Despite these limitations, we strongly believe that this study has made a case for the use of secondment as an important strategy towards bridging the divide between policy-makers and researchers.

The outcome of this study clearly suggests that secondment has a great potential of promoting evidence-informed policy-making. However, we agree with Grignon and colleagues,^28^ who noted that secondment requires substantial investment, and emphasis should be placed on high-level technical positions responsible for building systems, developing health workers, and strengthening government to translate policy into programs. We also subscribe to the suggestion of Hendrix-Jenkins et al^43^ that secondment should methodically and explicitly incorporate proven capacity development mechanisms such as coaching, mentoring, on-the-job training, or co-creation of strategies, models, processes, and/or tools. Undoubtedly secondment between research institutions and policy-making organizations as a mechanism that will promote evidence to policy link merits further consideration.

## Acknowledgments

This investigation received financial support from the UNCICEF/UNDP/World Bank/WHO Special Programme for Research and Training in Tropical Diseases (WHO/ TDR), (Reference 2015/B40427). Authors are grateful to all the policymakers, researchers and other stakeholders in MNCH in Nigeria who participated in this study.

## Ethical issues

The ethical clearance for this study was obtained from the EBSU Research Ethics Committee and approval was also obtained from the MoH. All work was performed according to the international ethical principles for medical research involving human subjects.^18^

## Competing interests

Authors declare that they have no competing interests.

## Authors’ contributions

Study conception and design by all authors. Data acquisition, analysis and interpretation by CJU, AEE, HCU, and CTE. Drafting of the manuscript by CJU. Critical revision of the manuscript for important intellectual content by AEE and HCU. Obtaining funding, administrative, technical, and material support by all authors.

## Authors’ affiliations

^1^African Institute for Health Policy & Health Systems, Ebonyi State University, Abakaliki, Nigeria. ^2^Department of Medical Microbiology/Parasitology, Faculty of Medicine, Ebonyi State University, Abakaliki, Nigeria. ^3^Department of Accountancy & Banking/Finance, Faculty of Management Sciences, Ebonyi State University, Abakaliki, Nigeria. ^4^National Obstetrics Fistula Centre, Abakaliki, Nigeria. ^5^Department of Paediatrics, Faculty of Medicine, Ebonyi State University, Abakaliki, Nigeria. ^6^Ebonyi State Ministry of Health, Abakaliki, Nigeria.

## Key messages

**Implications for policy makers**

- Secondment between the university and ministry of health in which researchers and policy-makers spend time in each other’s organization can facilitate institutional and individual collaboration.
- Evidence-informed policy-making can be greatly enhanced if policy-makers participate in research activities and researchers get involved in policy-making and implementation processes.

**Implications for the public**

Available evidence indicates that there is a wide gap between researchers and policy-makers and this is a major challenge to the development of evidence-based-policy. A secondment programme between institutions which generate research evidence and policy-making organizations, can bridge this gap and institutionalize the evidence-informed policy-making and practice, thereby ensuring that policies will be of greatest benefit to the public.
